# Incidence Trends for SARS-CoV-2 Alpha and Beta Variants, Finland, Spring 2021

**DOI:** 10.3201/eid2712.211631

**Published:** 2021-12

**Authors:** Ravi Kant, Phuoc Truong Nguyen, Soile Blomqvist, Mert Erdin, Hussein Alburkat, Maija Suvanto, Fathiah Zakham, Veera Salminen, Viktor Olander, Minna Paloniemi, Leena Huhti, Sara Lehtinen, Bruno Luukinen, Hanna Jarva, Hannimari Kallio-Kokko, Satu Kurkela, Maija Lappalainen, Hanna Liimatainen, Sari Hannula, Jani Halkilahti, Jonna Ikonen, Niina Ikonen, Otto Helve, Marianne Gunell, Tytti Vuorinen, Ilya Plyusnin, Erika Lindh, Pekka Ellonen, Tarja Sironen, Carita Savolainen-Kopra, Teemu Smura, Olli Vapalahti

**Affiliations:** University of Helsinki, Helsinki, Finland (R. Kant, P. Truong Nguyen, M. Erdin, H. Alburkat, M. Suvanto, F. Zakham, V. Salminen, V. Olander, H. Jarva, I. Plyusnin, T. Sironen, T. Smura, O. Vapalahti);; Finnish Institute for Health and Welfare (THL), Helsinki (S. Blomqvist, J. Halkilahti, J. Ikonen, N. Ikonen, O. Helve, E. Lindh, C. Savolainen-Kopra);; Fimlab Laboratories Ltd., Tampere, Finland (M. Paloniemi, L. Huhti, S. Lehtinen, B. Luukinen);; HUS Diagnostic Center, University of Helsinki and Helsinki University Hospital, Uusimaa, Finland (H. Jarva, H. Kallio-Kokko, S. Kurkela, M. Lappalainen, H. Liimatainen, T. Smura, O. Vapalahti);; Institute for Molecular Medicine Finland (FIMM), Helsinki (S. Hannula, P. Ellonen);; Turku University Hospital, Turku, Finland (M. Gunell, T. Vuorinen)

**Keywords:** COVID-19, coronavirus disease, epidemiology, Finland, infectious disease transmission, phylogeny, public health surveillance, respiratory infections, SARS-CoV-2, Alpha variant, Beta variant, severe acute respiratory syndrome coronavirus 2, vaccination, vaccines, viruses

## Abstract

Severe acute respiratory syndrome coronavirus 2 Alpha and Beta variants became dominant in Finland in spring 2021 but had diminished by summer. We used phylogenetic clustering to identify sources of spreading. We found that outbreaks were mostly seeded by a few introductions, highlighting the importance of surveillance and prevention policies.

Several new variants of severe acute respiratory syndrome coronavirus 2 (SARS-CoV-2) have emerged globally, most notably variants of concern Alpha (B.1.1.7) (*1*), Beta (B1.351) (*2*), Gamma (P.1) (*3*), and most recently, Delta (B.1.617.2). Each variant is thought to pose an increased public health risk compared with the earlier wild-type strains that were circulating in 2020 because of >1 epidemiologic characteristics, such as higher transmissibility (*4*), greater immune escape properties toward antibodies from previous SARS-CoV-2 infection (*5*), lower response to current vaccines (*6*), or more severe outcomes or increased mortality rates (*7*). Detecting and monitoring these novel variants is essential in SARS-CoV-2 surveillance.

## The Study

To assess the temporal epidemiologic dynamics among different variants of concern and identify spreading events and sources of SARS-CoV-2 cases detected in Finland, we began sequencing 400–1,000 virus samples per week collected during December 2020–May 2021 and analyzed the resulting genomes (n = 14,080), which are now available in the GISAID (https://www.gisaid.org) database. For quality control purposes, we removed all sequences with ≥2.0% gaps. 

We analyzed the resulting dataset (n = 9,160) with Pangolin (https://cov-lineages.org) (*8*) to identify lineages, from which we filtered Alpha and Beta variants for phylogenetic analyses. Each phylogenetic tree was computed from the filtered sequences and a global reference dataset consisting of 5 representative sequences, 1 sequence from the country of origin (England for Alpha, South Africa for Beta) and 4 randomly chosen from other countries containing the same lineage, for each date during December 2020–May 2021. The reference datasets included 841 genomes for Alpha variant and 775 genomes for Beta variant trees. We aligned sequences using MAFFT (https://mafft.cbrc.jp) (*9*) and removed gaps in the resulting alignments by trimming 50 characters from both the 5′ and 3′ ends. 

We then used the aligned sequences to compute the trees with a SARS-CoV-2–specific version of IQ-TREE 2 (*10*) using ModelFinder to identify and use the optimal nucleotide substitution model, performing 1,000 ultrafast bootstraps. We set the initial wild-type reference strain (GenBank accession no. NC_045512.2) as the outgroup. We assigned sequences to clusters using TreeCluster (*11*) based on an arbitrary branch length of 0.001 to identify major transmission chains. We collapsed clusters with ≤5 sequences for visualization purposes.

By May 2021, there had been 93,393 laboratory-confirmed SARS-CoV-2 infections reported in Finland (*12*); incidence peaks occurred in April and December 2020 and March 2021 (Appendix Figure 1, panel A). During this period, the weekly number of cases was as high as 4,900. National vaccinations began in late December 2020, and within 7 months, 3.5 million (62.8% of total population) persons had received first doses and 1.4 million (24.5% of total population) second doses (*13*). Seroprevalence remained low (<2%) until February 2021 (*14*) but increased because of growing vaccination coverage (Appendix Figure 1, panel B).

Throughout 2020, sequencing-based surveillance of the virus was conducted in the Hospital District of Helsinki and Uusimaa (HUS; Helsinki, Finland), which had the highest number of COVID-19 cases in the country (n = 21,742). Until December 18, 2020, only wild-type strains of SARS-CoV-2 had been detected, but the emergence of Alpha and Beta variants led to increased sequencing and sampling efforts at points of entry into Finland (i.e., airports, harbors, land border crossing sites) starting in week 51 of 2020. 

During December 2020–May 2021, a total of 14,080 SARS-CoV-2 genomes representing ≈20.4% of the PCR-confirmed SARS-CoV-2 infections (n = 65,921) were sequenced. During this period, the Alpha variant (5,370 total detections) comprised 58.6% of all cases, and its proportion in weekly counts rapidly increased from 3 (6.0%) of 50 in week 51 of 2020 to 602 (69.1%) of 871 in week 11 of 2021 (Appendix Figure 2). The highest proportion of Alpha variant cases was 240 (82.2%) of 292 detections in week 17. Beta variant incidence rose later and at a slower rate (1,049 total detections, 19.5% of all cases); the proportion in weekly case counts rose from 2 (1.7%) in week 2 of 2021 to 181 (23.1%) by week 12. The proportions of Alpha and Beta variants started to diminish in week 13. Only 1 Gamma variant case was recorded, in week 10, and the first Delta variant samples in Finland were collected during week 17. In addition, several variants of interest (*15*) were detected beginning in early January 2021: B.1.429 (2 detections), B.1.525 (25 detections), B.1.526 (1 detection), B.1.617.1 (6 detections), and P.2 (1 detection). Of the variants being monitored (*15*), 18 cases of AT.1 and 29 of B.1.1.318 lineages were detected during this period.

**Figure 1:**
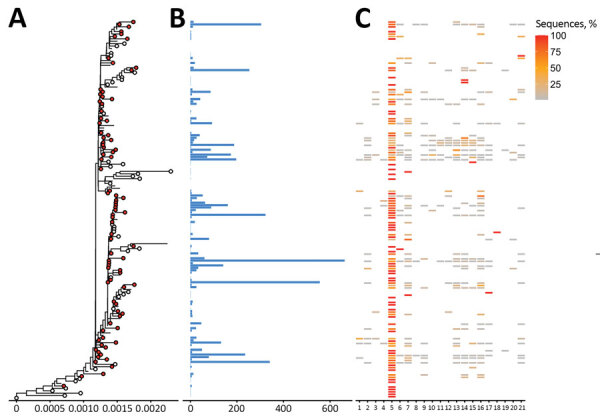
Phylogenetic tree of severe acute respiratory syndrome coronavirus 2 Alpha (B.1.1.7) variant clusters from Finland and sequence distribution. The tree (A) shows 86 clusters with ≥5 sequences (red circles), of which 84 contain 5,270 sequences sampled in Finland using TreeCluster, and 32 Finland singletons (white circles). The tree was constructed by using IQ-TREE 2 (*10*) with 1,000 ultrafast bootstraps. Each row in subsequent graphs is equivalent to a cluster and shows the number of sequences from Finland (B) and the proportion of sequences per region of Finland (C). Regions of Finland: 1, Åland Islands; 2, Central Finland Health Care District; 3, Central Ostrobothnia Hospital District; 4, East Savo Hospital District; 5, Hospital District of Helsinki and Uusimaa; 6, Hospital District of South Ostrobothnia; 7, Hospital District of Southwest Finland; 8, Kainuu Social and Health Care Joint Authority; 9, Kanta-Häme Hospital District; 10, Länsi-Pohja Healthcare District; 11, Lapland Hospital District; 12, North Karelia Hospital District; 13, North Ostrobothnia Hospital District; 14, North Savo Hospital District; 15, Päijät-Häme Hospital District; 16, Pirkanmaa Hospital District; 17, Satakunta Hospital District; 18, Social and Health Services in Kymenlaakso; 19, South Karelia Social and Health Care District; 20, South Savo Hospital District; 21, Vaasa Hospital District.

The clustering analysis of Alpha variants (Figure 1) showed 86 distinct clusters, of which 84 contained 5,270 sequences from Finland (57.5% of all sequences). The 13 largest clusters from Finland (total n = 3,669, 69.6%) had 132–663 sequences each. We detected 32 singletons (0.6% of Alpha detections) from Finland, suggesting that the epidemic was largely seeded from a few introductions, which aligns with the super-spreading properties of SARS-CoV-2 epidemiology. Most Alpha sequences were from the HUS district (n = 3,476, 64.7% of cases). We included all available high-quality sequences from random populations from Finland and thus included data from both mild and severe cases. However, a proportion of the samples from the HUS region came from points of entry into Finland and other hospital districts. The proportions of these imported samples varied over the sampling period depending on travel restrictions and hospitalized case-patients, which may have led to nonrandomized sampling from the HUS region.

**Figure 2:**
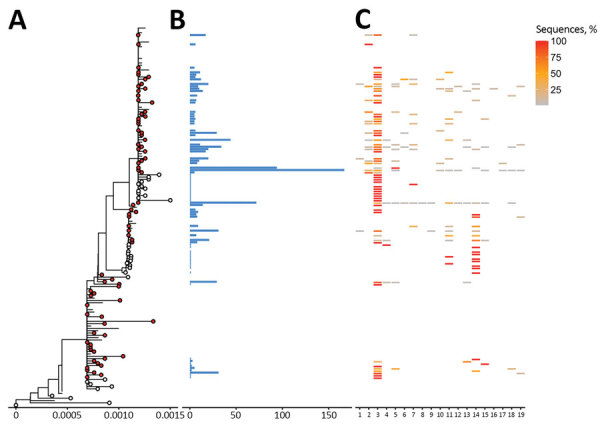
Phylogenetic trees of severe acute respiratory syndrome coronavirus 2 Beta (B1.351) variant clusters from Finland and sequence distribution. The tree (A) shows 76 clusters with ≥5 sequences (red circles), of which 48 contain 898 sequences sampled in Finland using TreeCluster (*11*), and 23 Finland singletons (white circles) from 33. The tree was constructed by using IQ-TREE 2 (*10*) with 1,000 ultrafast bootstraps. Each row in subsequent panels is equivalent to a cluster and shows the number of sequences from Finland (B) and the proportion of sequences per region of from Finland (C). Regions of Finland: 1, Central Finland Health Care District; 2, East Savo Hospital District; 3, Hospital District of Helsinki and Uusimaa; 4, Hospital District of South Ostrobothnia; 5, Hospital District of Southwest Finland; 6, Kainuu Social and Health Care Joint Authority; 7, Kanta-Häme Hospital District; 8, Länsi-Pohja Healthcare District; 9, Lapland Hospital District; 10, North Karelia Hospital District; 11, North Ostrobothnia Hospital District; 12, North Savo Hospital District; 13, Päijät-Häme Hospital District; 14, Pirkanmaa Hospital District; 15, Satakunta Hospital District; 16, Social and Health Services in Kymenlaakso; 17, South Karelia Social and Health Care District; 18, South Savo Hospital District; 19, Vaasa Hospital District,

Beta variants formed 76 distinct clusters, of which 56 contained 910 sequences from Finland (9.9% of all sequences from Finland) (Figure 2). We also identified 33 singletons, of which 23 were from Finland (2.2% of Beta detections). In total, there might have been 79 introductions from other countries, which seeded 1 major cluster (>100 Finland sequences) containing 167 sequences (15.9% of cases). Most Beta sequences were also from the HUS hospital district (n = 505, 48.1% of cases). Hospital district reports were based on data from the Finnish Institute for Health and Welfare (https://sampo.thl.fi), HUS, and Fimlab (https://fimlab.fi).

## Conclusions

Altogether, our study shows both Alpha and Beta variants emerging early and rapidly beginning in December 2020. Most (98.2% Alpha, 86.8% Beta) formed clusters, and only a small proportion (0.6% Alpha, 2.2% Beta) were singletons. Because the singletons represent a small fraction of the sequences and many were transmitted directly from travelers, it is likely that a few introductions were able to seed the epidemic.

The Alpha and Beta variants dominated detected SARS-CoV-2 cases, although at lower numbers for Beta, during early 2021. Despite the rapid emergence of these variants, their incidence fell sharply (Appendix Figure 1, panel A). Incidence in Finland has been low compared with other countries in Europe, permitting use of more moderately restrictive prevention measures. Incidence, and therefore seroprevalence, remained relatively low until vaccines became available. Practices and policies enacted in Finland, including frequent testing, contact tracing, isolation, quarantine, and other nonpharmaceutical interventions, helped effectively interrupt chains of transmission, and ongoing national efforts have resulted in most of the population of Finland receiving at least the first vaccine dose. These findings suggest that with proper surveillance and preventative measures, along with moderate restriction compliance, the spread SARS-CoV-2 could be mitigated effectively.

Appendix Additional information on SARS-CoV2 Alpha and Beta variants in Finland.
