# Supplementary material for: Incidence Trends for SARS-CoV-2 Alpha and Beta Variants, Finland, Spring 2021
Source: Emerg Infect Dis. 2021 Dec;27(12):3137–41. doi: 10.3201/eid2712.211631 (PMC8632157; doi:10.3201/eid2712.211631)
Supplement: Appendix — Additional information on SARS-CoV2 Alpha and Beta variants in Finland. [file 21-1631-Techapp-s1.pdf]

Article DOI: https://doi.org/10.3201/eid2712.211631

Incidence Trends for SARS-CoV2 Alpha and Beta Variants, Finland, Spring 2021

Appendix

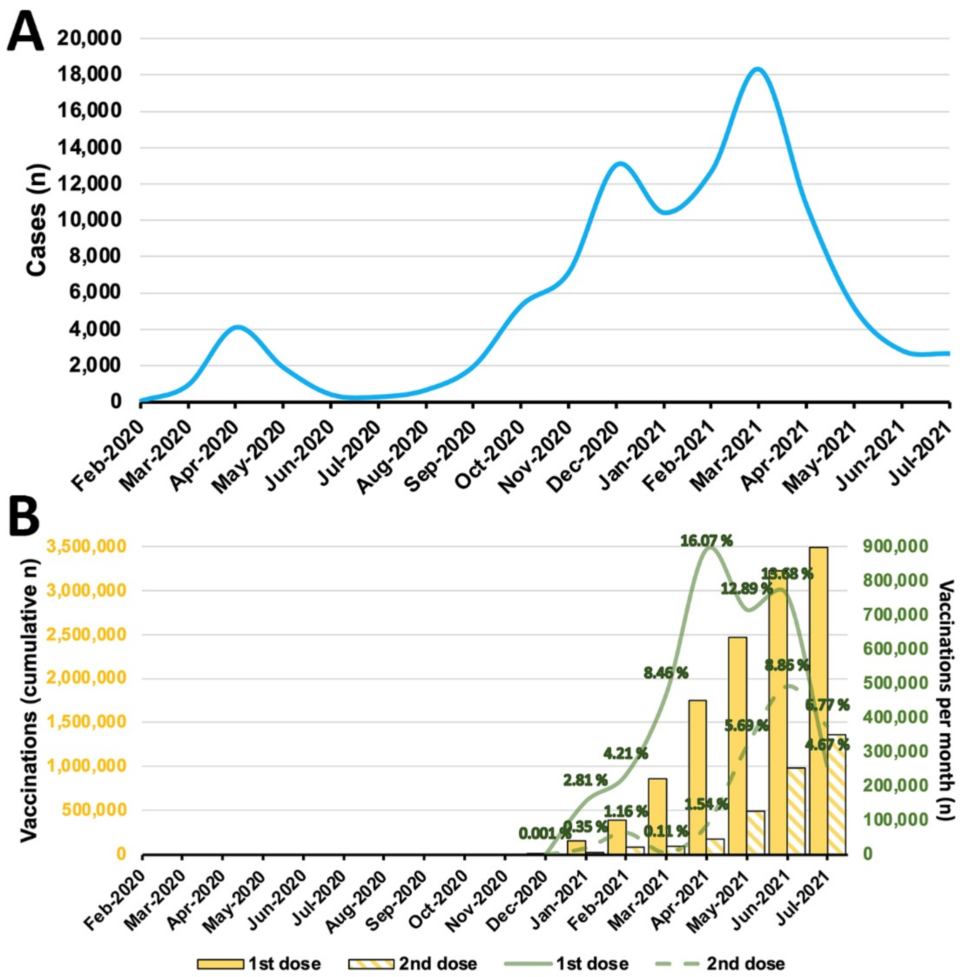

Appendix Figure 1. Monthly statistics for SARS-CoV-2 in Finland (February 2020–July 2021). A) 3 pandemic waves (April and December 2020, March 2021) since the virus arrived in Finland in early 2020. B) Finland national vaccination program began in December 2020. Yellow: total cumulative vaccinations green: monthly number of vaccinations. Monthly vaccination coverage among the population of Finland indicated in percentages. In total, ≈62.8% of the population had received the first dose and ≈24.5% had been injected with the booster dose as of July 2021. Values for SARS-CoV-2 cases and vaccinations were obtained from public records of Finnish Institute for Health and Welfare (https://sampo.thl.fi).

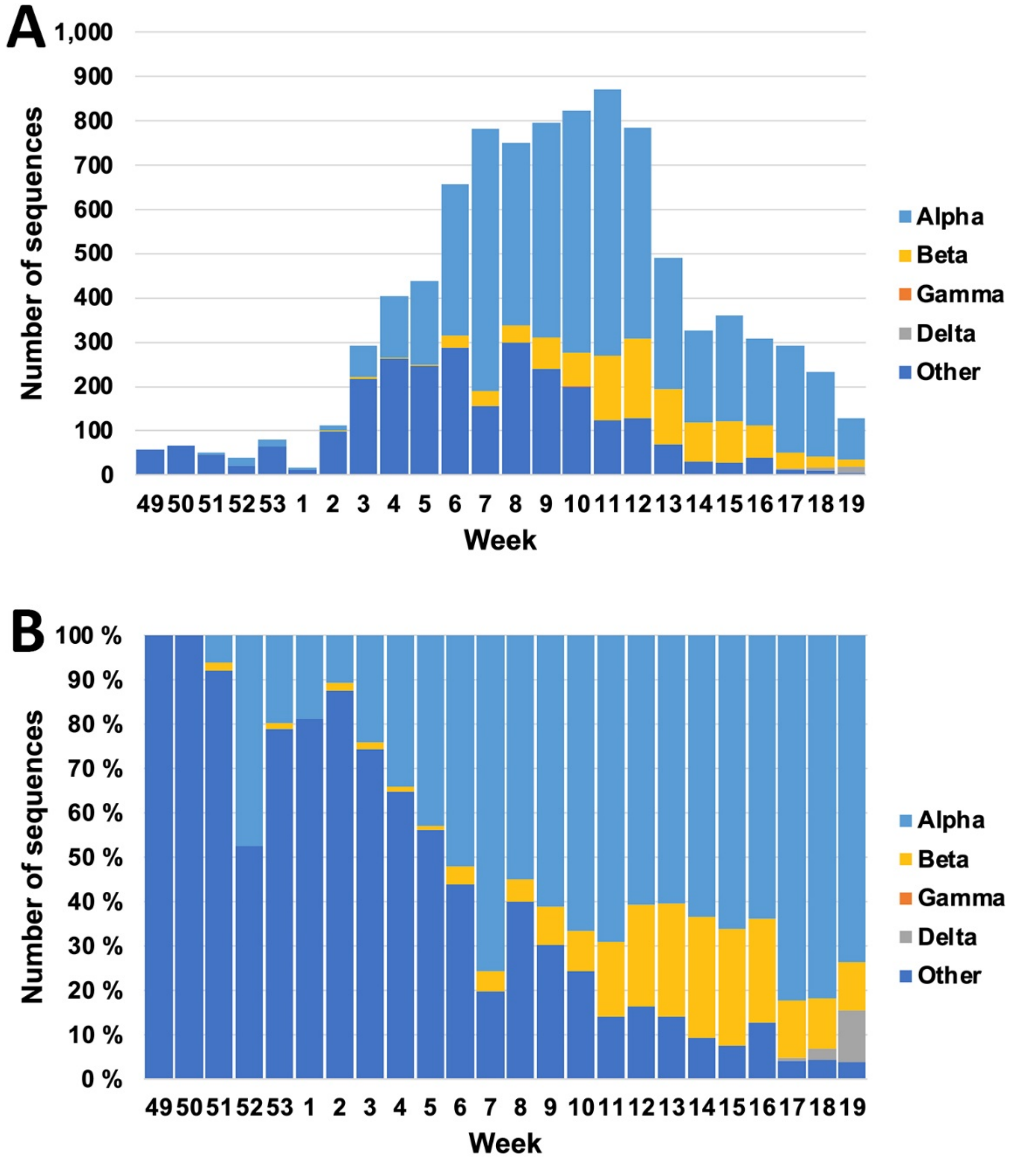

Appendix Figure 2. Weekly detection numbers of SARS-CoV-2 variants of concern during week 49 (December), 2020–week 19 (May), 2021 in Finland. A) Number of sequences; B) proportions (%).
